# A naphthalimide derivative exerts potent antiplatelet and antithrombotic activities without a bleeding tendency

**DOI:** 10.3389/fphar.2025.1541255

**Published:** 2025-06-24

**Authors:** Wan-Jung Lu, Jiun-Yi Li, Tzenge-Lien Shih, Ray-Jade Chen, Ting-Yu Chen, Wei-Ting Kao, Jen-Wei Liu, Hsueh-Hsiao Wang, Hsien-Yu Peng, Kuan-Hung Lin

**Affiliations:** ^1^ Department of Optometry, MacKay Medical College, New Taipei City, Taiwan; ^2^ Traditional Herbal Medicine Research Center, Taipei Medical University Hospital, Taipei, Taiwan; ^3^ Department of Medicine, MacKay Medical College, New Taipei City, Taiwan; ^4^ Department of Surgery, MacKay Memorial Hospital, Taipei, Taiwan; ^5^ Department of Chemistry, Tamkang University, New Taipei City, Taiwan; ^6^ Division of General Surgery, Department of Surgery, Taipei Medical University Hospital, Taipei, Taiwan; ^7^ Department of Surgery, School of Medicine, College of Medicine, Taipei Medical University, Taipei, Taiwan; ^8^ Institute of Biomedical Sciences, MacKay Medical College, New Taipei City, Taiwan

**Keywords:** GPVI, naphthalimide derivative, platelet activation, thrombus formation, antiplatelet agent

## Abstract

**Background:**

Bleeding is the inherent adverse effect of antiplatelet drugs that has limited their use in the prevention of secondary heart attack and stroke. Thus, finding a novel antiplatelet drug with antithrombotic activities while preserving hemostatic function remains a crucial issue. Here, we screened naphthalimide derivatives that we previously synthesized and identified a novel derivative compound **6**, which has a more potent antithrombotic effect and has no effect on bleeding cessation. This study is aimed to determine the antiplatelet mechanism of compound **6** and further test whether compound **6** is a safer and more potent antithrombotic agent.

**Methods:**

Platelet aggregation, flow cytometry and immunoblotting were used to determine the *in vitro* antiplatelet effect of compound **6**. The study of thrombus formation of mesenteric venules in mice was used to evaluate the antithrombotic effect of compound **6**.

**Results:**

Compound **6** selectively inhibited collagen-mediated platelet aggregation and markedly prevented thrombus formation without bleeding tendency. Compound **6** also inhibited glycoprotein VI (GPVI) downstream signaling, such as Fyn and Lyn, phospholipase C gamma 2, protein kinase C. Moreover, a surface plasmon resonance assay indicated that compound **6** may directly bind to GPVI, thereby interrupting the interaction of collagen and GPVI. Compound **6** also effectively attenuates collagen-induced granule release, calcium mobilization, and GPIIbIIIa activation.

**Conclusion:**

These findings indicate that compound **6** can selectively inhibit GPVI, eventually suppressing platelet activation and thrombus formation while preserving hemostasis. Compound **6** is a GPVI antagonist and safe antiplatelet agent. Compound **6** also has therapeutic potential for treating cardiovascular diseases.

## 1 Introduction

Platelets play a crucial role in physiological hemostasis and pathological thrombosis (Lindemann et al., 2007; Nieswandt et al., 2011). Insufficient platelet function may cause bleeding, and hyperactive platelets may be involved in atherosclerosis, which can lead to heart attack and stroke. Although many clinical antiplatelet agents have been used to treat patients with cardiovascular diseases, the inherent risk of bleeding of these antiplatelet agents has limited their use. Thus, developing novel antiplatelet agents that have little-to-no effect on hemostasis is crucial. Data from an *in vivo* study revealed key differences between hemostasis and thrombosis (McFadyen et al., 2018), indicating that a novel antiplatelet drug that prevents thrombus formation without affecting hemostasis can be developed. Several candidates, such as collagen receptor glycoprotein VI (GPVI), have been reported to achieve this goal (McFadyen et al., 2018). Previous reports have showed that patients with GPVI deficiency exhibit only mild bleeding, but their platelets fail to aggregate in response to collagen (Dutting et al., 2012). In an animal study, mice with genetic deletion and immunodepletion of GPVI exhibited protective effects against arterial thrombosis but moderate bleeding (Dutting et al., 2012). An anti–GPVI-Fab fragment (glenzocimab) and a dimeric GPVI-Fc fusion protein (Revacept), which can inhibit the interaction between platelets and collagen, were reported not to interfere with normal hemostasis (Ungerer et al., 2011; Lebozec et al., 2017). Additionally, fibrin and fibrinogen were also reported to activate GPVI and stabilize thrombi (Alshehri et al., 2015; Mammadova-Bach et al., 2015; Mangin et al., 2018). Such evidence indicates that targeting GPVI may be potential therapeutic strategy for developing antiplatelet or antithrombotic drugs.

**FIGURE 1:**
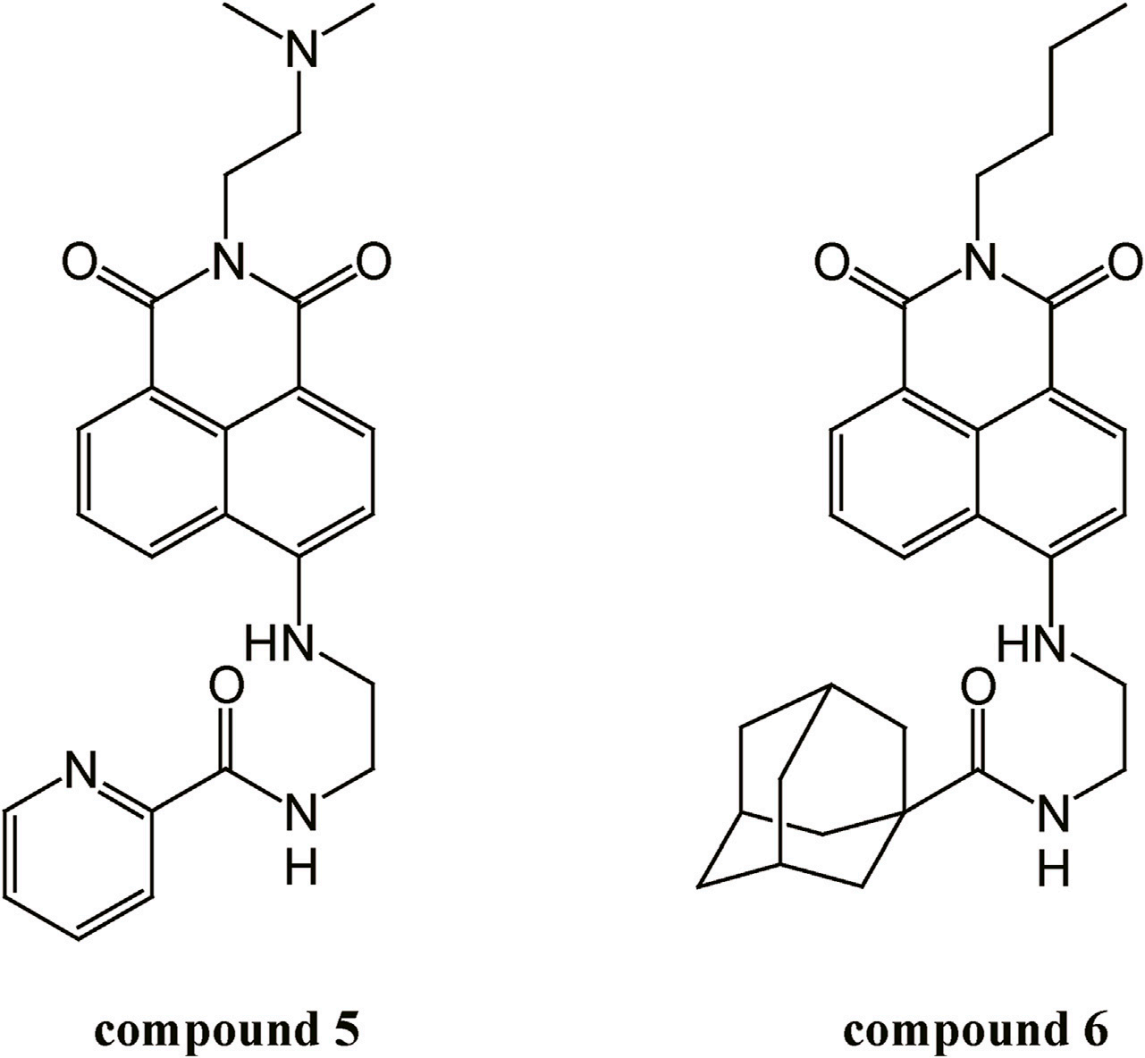
Structure of compounds **5** and **6**.

Naphthalimide derivatives possess multiple biological activities, including antitumor and anti-inflammatory effects (Kamal et al., 2013). Naphthalimide derivatives can easily intercalate into DNA and block cell division due to their flat structures (Kamal et al., 2013). Several naphthalimide derivatives were reported to possesses antitumor activities through suppressing DNA topoisomerase II and inducing lysosoml membrane permeabilization and apoptosis (Chen et al., 2010). The naphthalimide derivative 7 b effectively inhibited the activation of nuclear factor-kappaB (NF-κB) in lipopolysaccharide-stimulated RAW264.7 macrophages (Shao et al., 2013). Naphthalimides were also reported to exhibit antiviral activities through activation of REDD1 expression (Mata et al., 2011). In addition, naphthalimide derivatives was shown to act as free radical scavengers (Mata et al., 2011). We have previously demonstrated that a naphthalimide derivative compound **5** (Figure 1) targets GPVI and possesses potent antiplatelet and antithrombotic activities; however, it was associated with an exhibited bleeding tendency (Shih et al., 2021). Therefore, in this study, we focus on finding a novel naphthalimide derivative that does not have bleeding side effects and exhibits similar or better antiplatelet potency compared to compound **5**. Fortunately, in the present study, we reported a novel naphthalimide derivative compound **6** (Figure 1) that can inhibit platelet activation and thrombus formation while preserving hemostatic function and that is more selective in inhibiting collagen-induced platelet activation. Therefore, the present study analyzed the antiplatelet mechanisms of compound **6**.

## 2 Materials and methods

### 2.1 Materials

Collagen, thrombin, and U46619 were purchased from Chrono-Log (Havertown, PA, United States). The fluorescein sodium and luciferase/luciferin were purchased from Sigma Aldrich (St. Louis, MO, United States). Fura-2–acetoxymethyl (Fura-2 AM); anti–phospho-Lyn (Tyr^507^), anti–phospho-Fyn (Tyr^530^), anti–phospho-Akt (Ser^473^), and anti–Lyn polyclonal antibodies (pAbs) and anti–Fyn monoclonal antibodies (mAbs) were purchased from Abcam (Cambridge, United Kingdom). Anti-pleckstrin (p47) and anti–phospho-ERK1 (Thr^202^/Tyr^204^)/ERK2 (Thr^185^/Tyr^187^) pAbs were purchased from GeneTex (Irvine, CA, United States). Anti–phospho-(Ser) protein kinase C (PKC) substrate, anti–phospho-JNK (Thr^183^/Tyr^185^), anti–phospho-p38 mitogen-activated protein kinase (MAPK) (Thr^180^/Tyr^182^), and anti-JNK pAbs and anti-Akt, anti-p38 MAPK, and anti-ERK mAbs were purchased from Cell Signaling (Beverly, MA, United States). Horseradish peroxidase (HRP)-conjugated AffiniPure goat antirabbit, AffiniPure goat antimouse, and AffiniPure donkey antigoat immunoglobulin G (IgG) were purchased from Jackson ImmunoResearch (West Grove, PA, United States). Allophycocyanin (APC)-conjugated PAC-1 antibodies were purchased from Biolegend (San Diego, CA, United States). Hybond-P polyvinylidene difluoride membrane was purchased from GE Healthcare Life Sciences (Buckinghamshire, United Kingdom). A SuperLight Chemiluminescent HRP kit was purchased from Bionovas (Toronto, Canada).

### 2.2 Synthesis of compound 6

Compound **6** was synthesized as previously described (Tung et al., 2020). Compound **6** consists of a naphthalimide framework. C4 position of compound **6** is linked with an ethylenediamine group that is coupled with a 1-adamantane carbonyl moiety. Compound **6** was dissolved in dimethyl sulfoxide (DMSO) and stored at 4°C until use.

### 2.3 Preparation of human platelet suspensions

This study received approval from the Taipei Medical University–Joint Institutional Review Board (TMU-JIRB-No. N202003148) and adhered to the principles of the *Declaration of Helsinki*. Informed consent was obtained from all volunteers before their participation. Human platelets were washed and prepared according to previously established methods (Lu et al., 2019). Whole blood was collected from healthy participants who had not taken any medications, including nonsteroidal anti-inflammatory drugs or aspirin, in the preceding 2 weeks. The blood was drawn into polypropylene tubes containing an acid citrate–dextrose solution (9:1, v/v). After mixing, the samples were centrifuged at 120 *g* for 10 min. The upper layer of platelet-rich plasma was collected and supplemented with prostaglandin E_1_ and heparin. Following further centrifugation at 500 *g* for 10 min, the platelet pellets were washed twice. The platelets were then resuspended in Tyrode’s solution containing 3.5 mg/mL bovine serum albumin (B.S.A.) to create platelet suspensions. The final Ca^2+^ concentration of the platelet suspensions (3.6 × 10^8^ cells/mL) was 1 mM.

### 2.4 Platelet aggregation

Platelet aggregation was measured using a lumi-aggregometer (Payton, Scarborough, Canada) through the turbidimetric method (Lu et al., 2019). Human platelet suspensions (3.6 × 10^8^ cells/mL) were treated with compound **6** (1–50 μM) or an isovolumetric solvent control (0.1% DMSO) for 3 min. Subsequently, various agonists were added and platelet aggregation was then recorded for an additional 6 min.

### 2.5 Animals

Male ICR mice (weighing 20–25 g and aged 5–6 weeks) were purchased from BioLasco (Taipei, Taiwan). The animal use protocol for this project was approved by the Affidavit of Approval of Animal Use Protocol–Taipei Medical University (LAC-2020–0074). All animal experiments were conducted in accordance with the Guide for the Care and Use of Laboratory Animals, Eighth Edition (2011).

### 2.6 Thrombus formation in the mesenteric microvessels of mice

The mice were anesthetized with an oxygen–air mixture containing 3% isoflurane at a gas flow rate of 1.5–2 L/min. A bolus dose (bolus volume: 20 μL) of compound **6** (0.3 mg/kg, 0.6 mg/kg, or 1.2 mg/kg), DMSO (solvent control; 1.1 g/kg), or aspirin (20 mg/kg; positive control) was intravenously administered through the tail vein before the administering sodium fluorescein (15 mg/kg). The small intestinal segments were placed on a transparent culture dish, and the mesenteric vessels were observed under a microscope. Venules (20–30 μm) were selected and irradiated with light (wavelength <520 nm) to induce endothelial damage, causing thrombus formation and subsequent vessel occlusion. The time required to occlude a microvessel was recorded (Lin et al., 2013). A formula for dose translation based on the bovine serum albumin was used to calculate the appropriate dose for use in the mice (Reagan-Shaw et al., 2008). Reagan-Shaw et al. (2008) reported that the animal dose should not be extrapolated to a human equivalent dose (HED) by a simple conversion based on body weight. They suggested using the bovine serum albumin (BSA) normalization method and this method correlates well across several mammalian species with several parameters of biology. The formula for dose translation based on BSA is as follows: HED (mg/kg) = Animal dose (mg/kg) multiply by Animal *Km*/Human *Km*. Mouse *Km* is 3 and Human *Km* is 37.

### 2.7 Collagen/epinephrine-induced pulmonary embolism in mice

Pulmonary embolism was induced using collagen and epinephrine in 3 male and 3 female ICR mice according. Briefly, mice were intravenously injected with a bolus dose of DMSO (1.1 g/kg, solvent control), compound **6** (1.2 mg/kg), or aspirin (20 mg/kg, positive control). Subsequently, a mixture of collagen (0.6 mg/kg) and epinephrine (0.2 mg/kg) was administered via the tail vein. When respiration became severely weakened but the heart was still beating, 0.5 mL of Evans blue solution (1% in saline) was injected into the heart. The lungs were then excised and photographed. Mouse mortality was monitored for 24 h, and all surviving mice were euthanized immediately after the experiment. Each group consisted of six animals.

### 2.8 FeCl_3_-induced mesenteric artery thrombosis in mice

Mice were anesthetized with an oxygen–air mixture containing 3% isoflurane at a gas flow rate of 1.5–2 L/min. To label platelets, they were injected with the fluorescent dye rhodamine 6G (0.6 mg/kg). 10 min before FeCl_3_ treatment, mice were intravenously administered DMSO (1.1 g/kg, solvent control), compound **6** (1.2 mg/kg), or aspirin (20 mg/kg, positive control). The mesenteric arteries were surgically exposed and injured by applying a filter paper saturated with 30% FeCl_3_ for 5 min to induce thrombosis. Thrombus formation was then continuously monitored using a fluorescence microscope (Olympus, Tokyo, Japan).

### 2.9 Tail-bleeding assay

For the tail-bleeding assay, the mice were anesthetized with an oxygen–air mixture containing 3% isoflurane at a gas flow rate of 1.5–2 L/min. A bolus dose of compound **6** (0.3 mg/kg, 0.6 mg/kg, or 1.2 mg/kg), DMSO (solvent control; 1.1 g/kg), or aspirin (20 mg/kg; positive control) was intraperitoneally administered for 30 min. A 3-mm incision was then made at the tail tip to induce bleeding. The bleeding tail stump was immediately immersed in saline, and the bleeding time was recorded. Bleeding time was defined as the interval from the start of bleeding until no bleeding was observed for at least 10 s (Lien et al., 2017). A formula for dose translation based on the bovine serum albumin was used to calculate the dose for use in the mice (Reagan-Shaw et al., 2008).

### 2.10 Western blotting

Western blotting was performed as described previously (Lien et al., 2017). Human platelet suspensions (3.6 × 10^8^ cells/mL) were pretreated with compound **6** (2.5 and 5 μM) or 0.1% DMSO for 3 min and then treated with collagen for 6 min. After centrifugation, the platelet pellets were immediately resuspended in lysis buffer (200 μL) for 1 h. The supernatants were collected after centrifugation at 5,000 *g* for 5 min, and the protein extracts (80 μg) were subjected to 8%–12% sodium dodecylsulfate–polyacrylamide gel electrophoresis. The separated proteins were then electrotransferred onto a polyvinylidene fluoride membrane through a semidry transfer (Thermo Fisher, Waltham, MA, United States). The membrane was blocked with TBST (10 mM Tris-base, 100 mM NaCl, and 0.01% Tween-20) containing 5% B.S.A. for 1 h. After being washed three times, the membrane was incubated with various specific primary antibodies (1:1,000). Subsequently, the membrane was incubated with HRP-conjugated antibodies (1:5,000) for 1 h. Immunoreactive bands were developed using an electrochemiluminescence kit and analyzed using Celvin S (Biostep, Burkhardtsdorf, Germany).

### 2.11 Surface plasmon resonance

Surface plasmon resonance (SPR) analysis was performed using an OpenSPR instrument (Nicoya, Kitchener, Canada). A nitrilotriacetic acid (NTA) sensor chip was installed in the OpenSPR instrument. Running buffer (phosphate-buffered saline) was pumped at the maximum flow rate, and 80% isopropanol was injected to remove any bubbles. Subsequently, 200 μL of a 200 mM imidazole solution was injected to prime the sensor surface. After rinsing the chip with running buffer, 200 μL of a 40 mM NiCl_2_ solution was used to activate the NTA chip. After 5 min of interaction time, his-tagged recombinant GPVI proteins were bound to the NTA chip by perfusing the GPVI protein solution at a flow rate of 20 μL/min. Solutions of compound **5** or compound **6** (0.1 and 1 μM) were then injected. The equilibrium dissociation constant (KD) was determined using the OpenSPR system (Nicoya).

### 2.12 Molecular docking

Crystal structure of human platelet GPVI (PBD: 2GI7) from Protein Data Bank and the structure of compound **6** from Chemical Sketch Tool were downloaded. Then, the possible binding site was predicted through molecular docking using AutoDock 4. The images were generated using ChimeraX, AutoDock 4, and Discovery Studio Visualizer, respectively.

### 2.13 Adenosine triphosphate release and calcium mobilization in human platelet suspensions

Adenosine triphosphate (ATP) release and calcium mobilization were assessed as previously described (Shih et al., 2021). Luciferase–luciferin and Fura-2 AM were used to detect ATP release and calcium mobilization, respectively. The intensity of luminescence (indicating ATP release) and the fluorescence ratio (340 nm/380 nm) for calcium mobilization were measured using a Hitachi Spectrometer F-7000 (Tokyo, Japan) following the manufacturer’s instructions.

### 2.14 Flow cytometry

Flow cytometry was performed as previously described (Lien et al., 2017). After 20 min of collagen stimulation, the human platelets were fixed and labeled with APC-conjugated PAC-1 antibodies for 30 min to detect the level of GPIIb–IIIa activation. After centrifugation and washing, the platelets were suspended in 1 mL of phosphate-buffered saline and analyzed using a CytoFLEX flow cytometer (Beckman Coulter Life Sciences, Indianapolis, IN, United States). In the flow cytometry setup, platelets were gated using forward scatter and side scatter, and the number of events was set to stop at 10,000 counts. All experiments were performed at least three times to ensure reliable results.

### 2.15 Statistical analysis

The data were analyzed using an analysis of variance, followed by a Newman–Keuls test for *post hoc* analysis. Results were expressed as means ± standard errors of the mean (SEM). A *p* value of less than 0.05 was considered statistically significant.

## 3 Results

### 3.1 Compound 6 has potent antiplatelet and antithrombotic effects without significant bleeding

**FIGURE 2:**
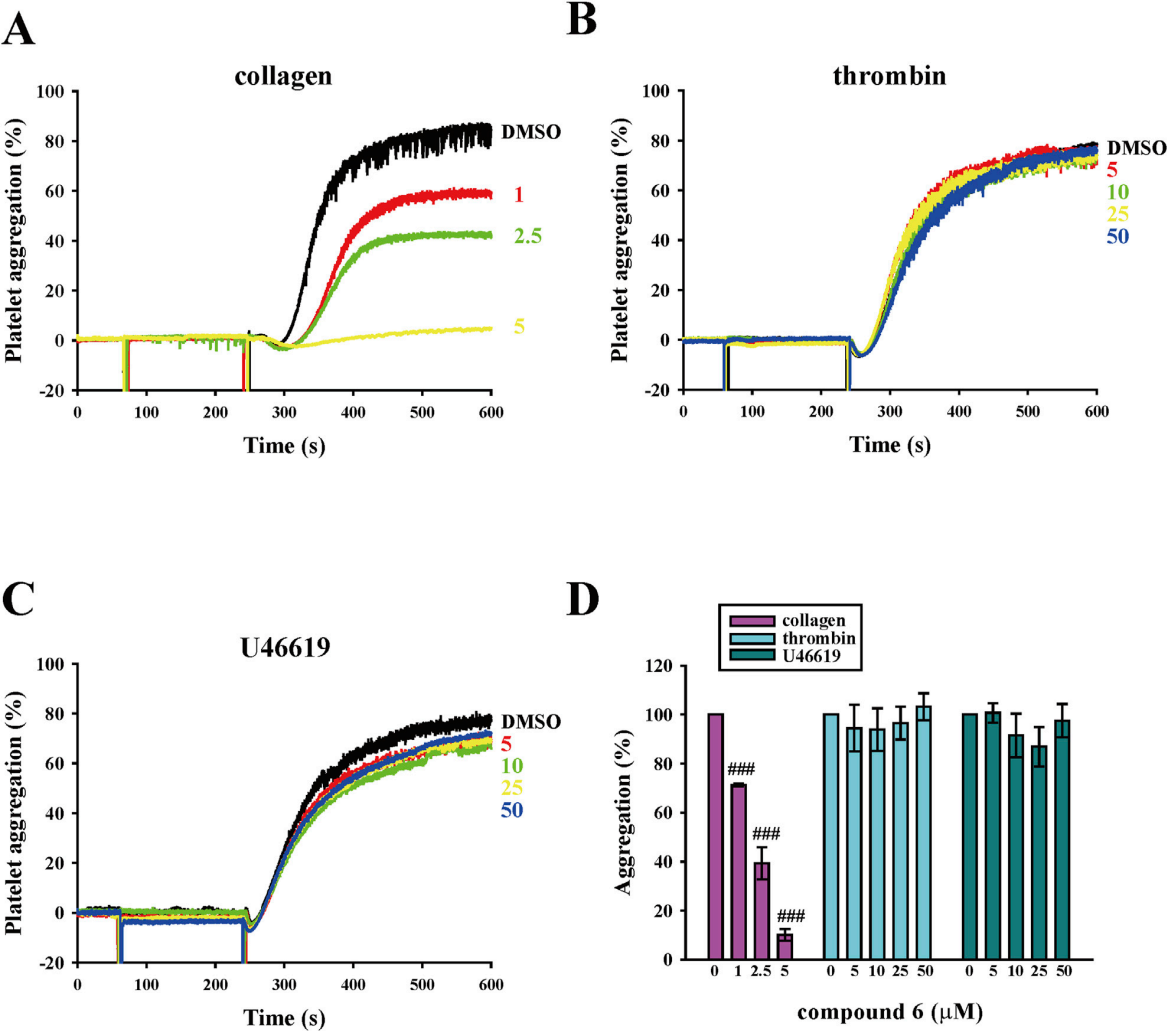
Effects of compound **6** on human platelet aggregation triggered by various agonists. **(A–C)** Washed platelets (3.6 × 10^8^ cells/mL) were treated with compound **6** (1–50 μM) or dimethyl sulfoxide (DMSO; solvent control) before adding 1 μg/mL collagen **(A)**, 0.02 U/mL thrombin **(B)**, and 1 μM U46619 **(C)**. Panels A–C present the curve of platelet aggregation, and panel D presents the statistical analysis of panels A–C. Data **(D)** are presented as means ± SEM (*n* = 4). ###*p* < 0.001 compared with the DMSO group.

We previously reported that a compound with naphthalimide moiety has antiplatelet and antithrombotic properties. However, the compound **5** that we synthesized demonstrated a bleeding tendency (Shih et al., 2021). Thus, we further screened the naphthalimide derivatives that we synthesized to identify those with more potent antiplatelet and antithrombotic activities that preserved hemostasis. In the platelet aggregation assay using human platelet suspensions, we discovered that compound **6** is potent and selective to inhibit collagen-induced platelet aggregation than compound **5**. As presented in Figures 2A,D, the data revealed that compound **6** could inhibit collagen-induced platelet aggregation in a concentration-dependent manner (1–5 μM). In addition, at the same concentration (5 μM), compound **6** showed better antiplatelet effects than compound **5** (Supplementary Figure S1). The IC50 of compound **6** was approximately 2.5 μM, indicating it had a better potency than compound **5** did (IC50 = 6.5 μM) (Shih et al., 2021). Moreover, unlike compound **5**, compound **6** (5–50 μM) did not significantly affect thrombin-, U46619, and ADP-induced platelet aggregation (Figures 2B–D; Supplementary Figure S2). In addition, our results show that compound **6** can inhibit convulxin-induced platelet aggregation, although at higher concentrations (10–50 μM) compared to those used in the collagen-induced platelet aggregation (Supplementary Figure S7). This difference may be attributed to the exceptionally high binding affinity of convulxin for GPVI (in the picomolar to low nanomolar range) (Horii et al., 2009). Thus, compound **6** is more potent and selective to inhibit collagen-induced platelet aggregation than compound **5** is.

**FIGURE 3:**
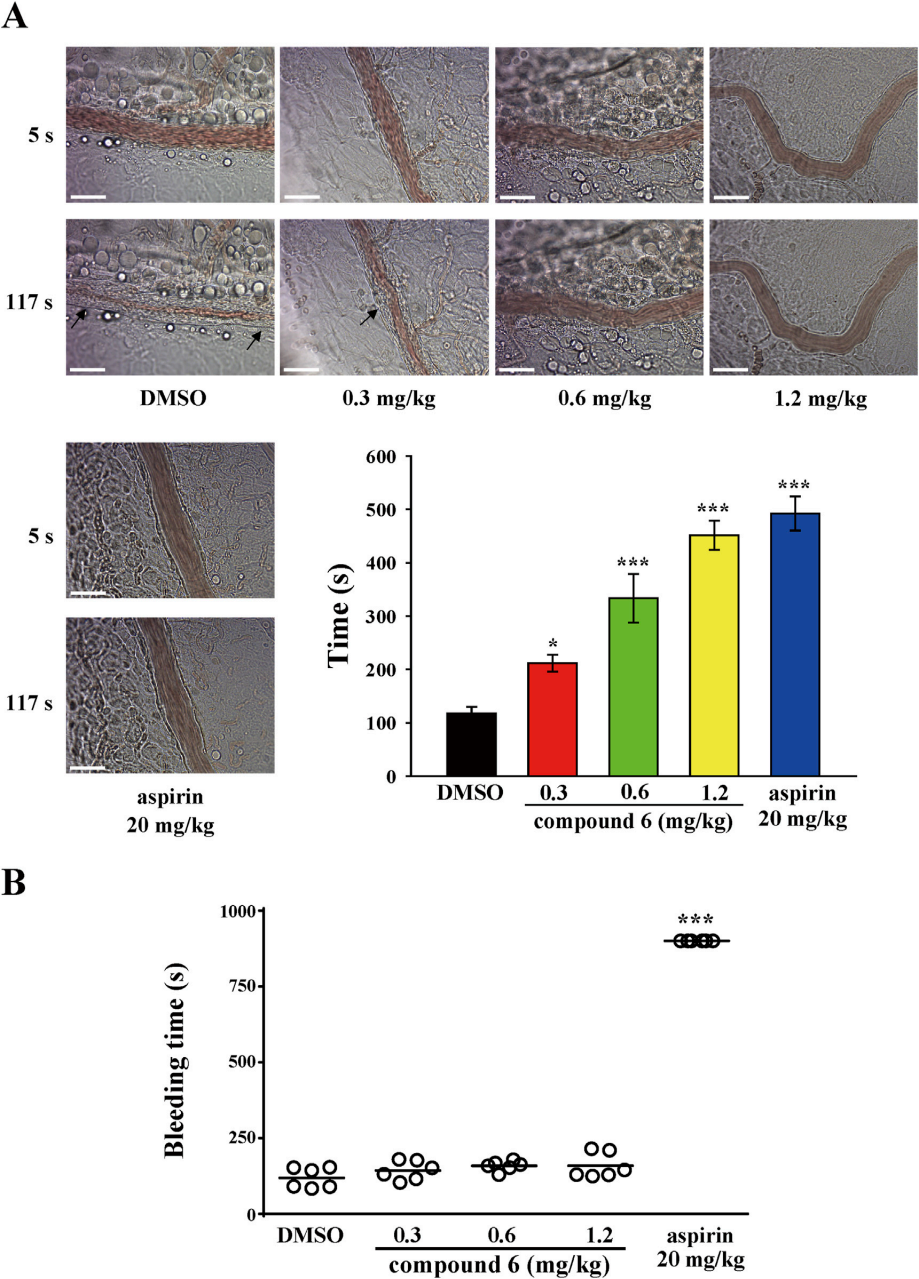
Effects of compound **6** on thrombosis and hemostasis in mice. Mice were treated with compound **6** (0.3, 0.6, or 1.2 mg/kg), DMSO (solvent control), or aspirin (20 mg/kg; positive control) via intravenous route for 10 min. **(A)** The microthrombus formation (arrows) in mesenteric venules. Scale bar = 30 μm. **(B)** The bleeding time was recorded after cutting the tail until no bleeding sign was observed for at least 10 s. Each point on the plot indicates a mouse (*n* = 6). Data **(A,B)** are presented as means ± SEM (*n* = 6). *p < 0.05 and ***p < 0.001 compared with the DMSO group.

**FIGURE 4:**
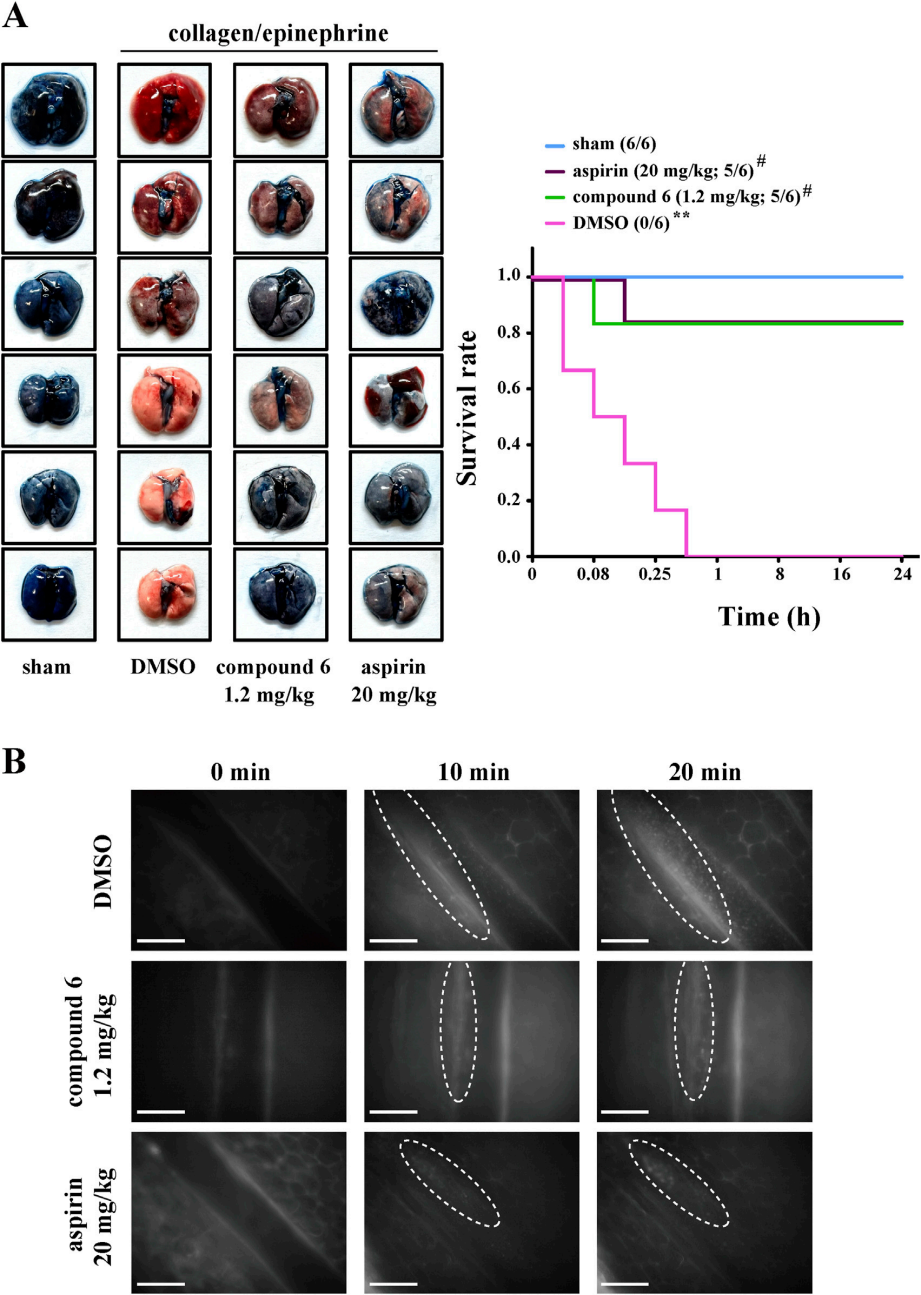
Effects of compound **6** on collagen/epinephrine-induced pulmonary thrombosis and FeCl_3_-induced thrombosis in mesenteric artery. Mice were intravenously administered with DMSO (solvent control), compound **6** (1.2 mg/kg), or aspirin (20 mg/kg, positive control) for 10 min **(A)** Mice were injected with collagen/epinephrine to induce pulmonary embolism, which was detected through staining with Evans blue (left panel). The survival rate was recorded for 24 h (right panel). Data are presented as means ± SEM (*n* = 6). **p < 0.01 compared with the sham group. #*p* < 0.05 compared with the DMSO (solvent control) group. **(B)** FeCl_3_-induced thrombosis in mesenteric artery as observed under real-time fluorescent microscopy. Thrombus was observed for the indicated time (0, 10, and 20 min). Scale bar = 100 µm.

For the thrombotic mouse model, the data revealed that compound **6** can dose-dependently (0.3, 0.6, or 1.2 mg/kg) delay the occlusion time in fluorescein sodium–mediated thrombus formation (arrows) in mesenteric microvessels (DMSO, 117.7 ± 12.4 s; 0.3 mg/kg, 211.5 ± 15.8 s; 0.6 mg/kg, 333.5 ± 45.6 s; 1.2 mg/kg, 451.5 ± 27.4 s). The aspirin group (20 mg/kg) was used as a positive control showing a good antithrombotic activity (492.2 ± 31.9 s) (Figure 3A). We previously reported that compound **5** inhibits thrombus formation at a dose of 2.3 mg/kg but not 1.2 mg/kg (Shih et al., 2021). These observations indicate that compound **6** exerts more potent antithrombotic effects than compound **5** does. In addition, in the pulmonary thrombotic model, collagen and epinephrine were used to induce lung thrombosis, which was visualized using Evans blue staining of the entire lung. The data showed that compound **6** could prevent collagen/epinephrine-induced pulmonary thrombosis and improve the survival rate (Figure 4A) relative to DMSO group. In the mesenteric artery thrombotic model (Figure 4B), FeCl_3_ was used to induce thrombosis. Thrombus formation was visualized by monitoring the accumulation of rhodamine 6G-labeled platelets under fluorescence microscopy. The data showed that compound **6** also delayed platelet accumulation on the injured vessels relative to DMSO group. Moreover, the tail-bleeding assay revealed that compound **6** did not significantly affect the duration of tail-bleeding (Figure 3B). These findings indicated that compound **6** exerts a more potent antithrombotic effect without a bleeding tendency. Therefore, compound **6** may be a safer and more potent antithrombotic agent than compound **5**. In subsequent experiments, we identified the mechanism underlying the antiplatelet effect of compound **6**.

### 3.2 Compound 6 inhibits GPVI signaling in human platelet suspensions

**FIGURE 5:**
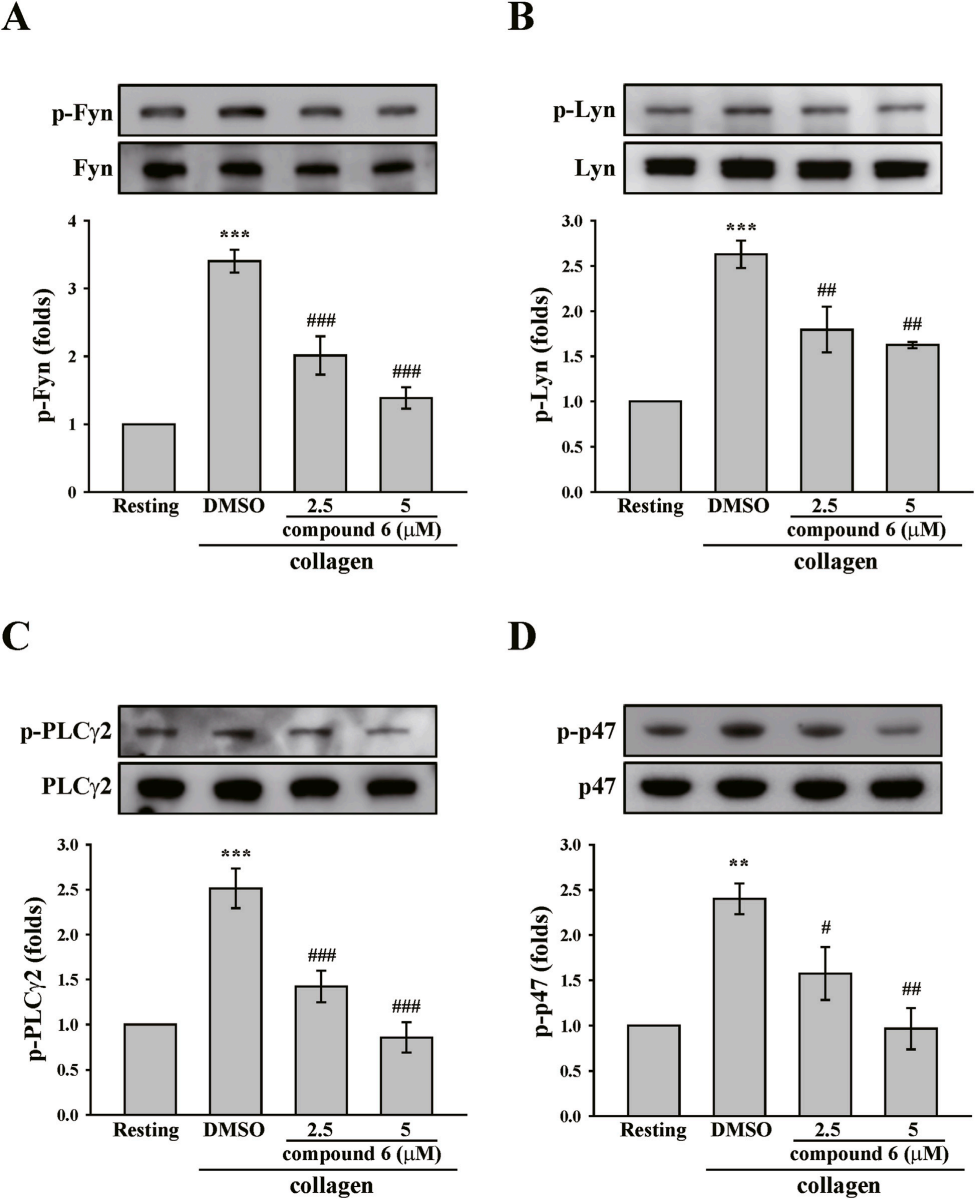
Effects of compound **6** on the phosphorylation of Fyn, Lyn, PLCγ2, and PKC substrates (p47) induced by collagen in human platelets. **(A–D)** Washed platelets (3.6 × 10^8^ cells/mL) were treated with compound **6** (2.5 and 5 μM) or DMSO before adding collagen (1 μg/mL). Proteins of these samples were separated by Western blotting. These targets (Fyn, Lyn, PLCγ2, and p47) were detected using specific antibodies. Data **(A–D)** are presented as means ± SEM (*n* = 5). **p < 0.01 and ***p < 0.001 compared with the resting group. #*p* < 0.05, ##*p* < 0.01, and ###*p* < 0.001 compared with the DMSO (solvent control) group.

The clustering of collagen receptor GPVI has been reported to induce transautophosphorylation of the activation loop tyrosine residue and maximal activation of the Src family kinases (Lyn and Fyn). These kinases then phosphorylate downstream effectors such as phospholipase C (PLC)-γ2 and PKC, propagating the signal (Senis et al., 2014; Estevez and Du, 2017). Thus, we determined the effect of compound **6** on GPVI signaling. As indicated in Figures 5A,B, collagen significantly induced Lyn and Fyn phosphorylation, which could be reversed using compound **6** (2.5 and 5 μM). Compound **6** also significantly reduced the collagen-induced phosphorylation of PLCγ2 and the substrate of PKC (p47 proteins), which is an indicator of PKC activation (Figures 5C,D).

**FIGURE 6:**
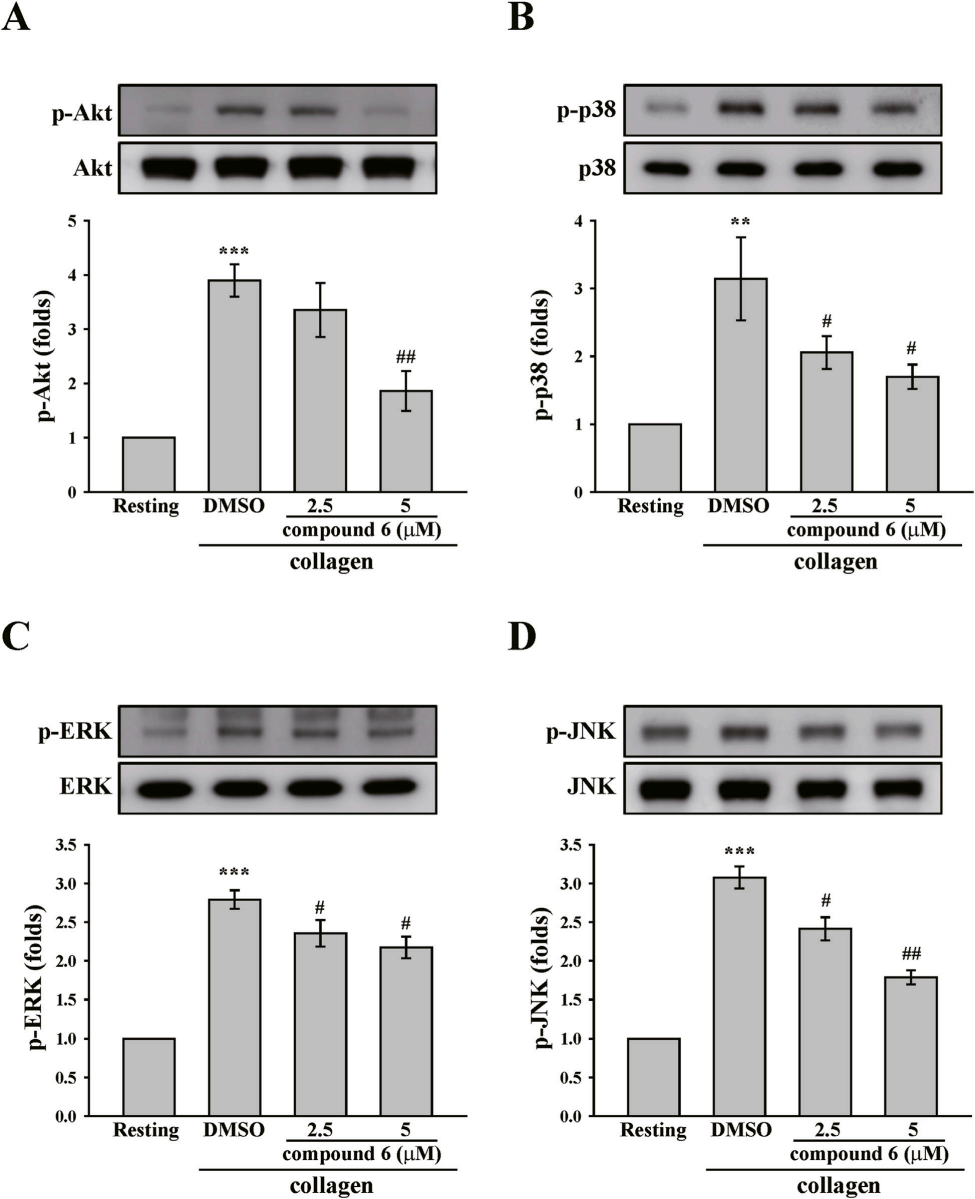
Effects of compound **6** on the phosphorylation of Akt, and MAPKs induced by collagen in human platelets. Washed platelets (3.6 × 10^8^ cells/mL) were treated with compound **6** (2.5 and 5 μM) or DMSO before adding collagen (1 μg/mL). Proteins of these samples were separated by Western blotting. Akt **(A)**, p38 MAPK **(B)**, ERK **(C)**, and JNK **(D)** were detected using specific antibodies. Data **(A–D)** are presented as means ± SEM (*n* = 4). **p < 0.01 and ***p < 0.001 compared with the resting group. #*p* < 0.05 and ##*p* < 0.01 compared with the DMSO (solvent control) group.

Akt and MAPKs, including ERK, p38 MAPK, and JNK, are downstream effectors of GPVI signaling (Li et al., 2010). These signaling molecules were also identified. The data revealed that collagen can induce considerable phosphorylation of Akt and MAPKs. Moreover, the phosphorylation of Akt and MAPKs could be diminished by compound **6** (5 μM) (Figure 6). Collectively, these findings suggest that compound **6** can inhibit GPVI signaling.

### 3.3 Compound 6 directly interferes with GPVI

**FIGURE 7:**
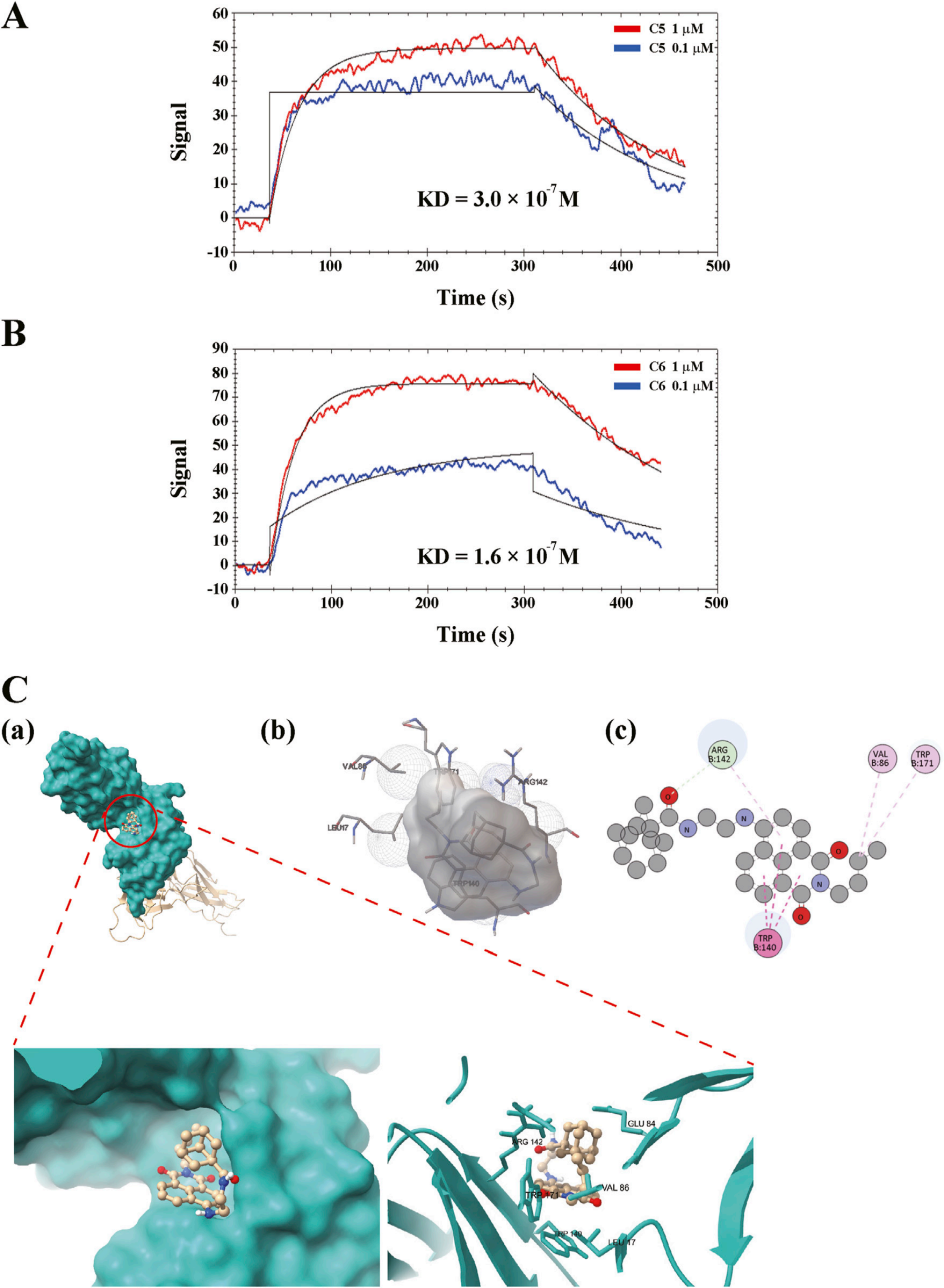
Direct binding of compound **6** on GPVI. The recombinant GPVI proteins were coated on the nitrilotriacetic acid sensor chip, followed by perfusing the solutions of **(A)** compound **5** (**C5**, 0.1 and 1 μM) or **(B)** compound **6** (**C6**, 0.1 and 1 μM) on the chip. Equilibrium dissociation constant (KD) were determined using the OpenSPR system. **(C)** The prediction of binding site of ligand and receptor. 3D **(A,B)** and 2D **(C)** representations of the binding interaction between compound **6** and GPVI using ChimiraX, Autodock 4, and Discovery Studio Visualizer, respectively.

We tested whether compound **6** could directly interfere with GPVI and verified whether compound **6** had a better binding affinity on GPVI than compound **5** did. An SPR binding assay was performed. First, recombinant human GPVI was coated on the NTA sensor chip. Solutions of compound **5** and compound **6** (0.1 and 1 μM) were then perfused on the chip, and the KD was determined using the OpenSPR system (Nicoya Lifesciences). The data revealed that compound **6** bound to GPVI in a concentration-dependent manner, which indicated a higher and better binding affinity (KD = 1.6 × 10^−7^ M) than that of compound 5 (KD = 3.0 × 10^−7^ M) (Figures 7A,B).

On the other hand, the possible binding site was predicted through molecular docking using AutoDock 4 (Figure 7C). The images were generated using ChimeraX, AutoDock 4, and Discovery Studio Visualizer, respectively. Simulation results indicated that hydrophobic interactions may play a crucial role in the interaction between the compound **6** and GPVI, suggesting that the shape of compound **6** must fit more precisely within the binding pocket, thereby enhancing its selectivity for GPVI. The molecular docking analyses predict that compound **6** can interact with GPVI D1/D2 domain that is proximal to the trowaglerix-binding domain (Chang et al., 2017), away from the collagen-binding domain. Trowaglerix venom polypeptides was reported to act as a novel antithrombotic agent against GPVI. Actually, crystals of GPVI complexed with the inhibitory nanobody, Nb2, reveal significant flexibility within the D1-D2 region consistent with possible allosteric modulation of ligand binding (Mangin et al., 2023). Moreover, D2 domain of GPVI has been confirmed to regulate ligand binding to D1 domain and prevent interaction of GPVI with collagen and fibrin (ogen) (Mangin et al., 2023). GPVI-fibrin (ogen) were also play a crucial role in stabilizing thrombi (Alshehri et al., 2015; Mammadova-Bach et al., 2015; Mangin et al., 2018). However, whether compound **6** can interfere with the interaction of GPVI-collagen or -finbin (ogen) remains to be clarified.

### 3.4 Compound 6 attenuates platelet activation events in human platelet suspensions

**FIGURE 8:**
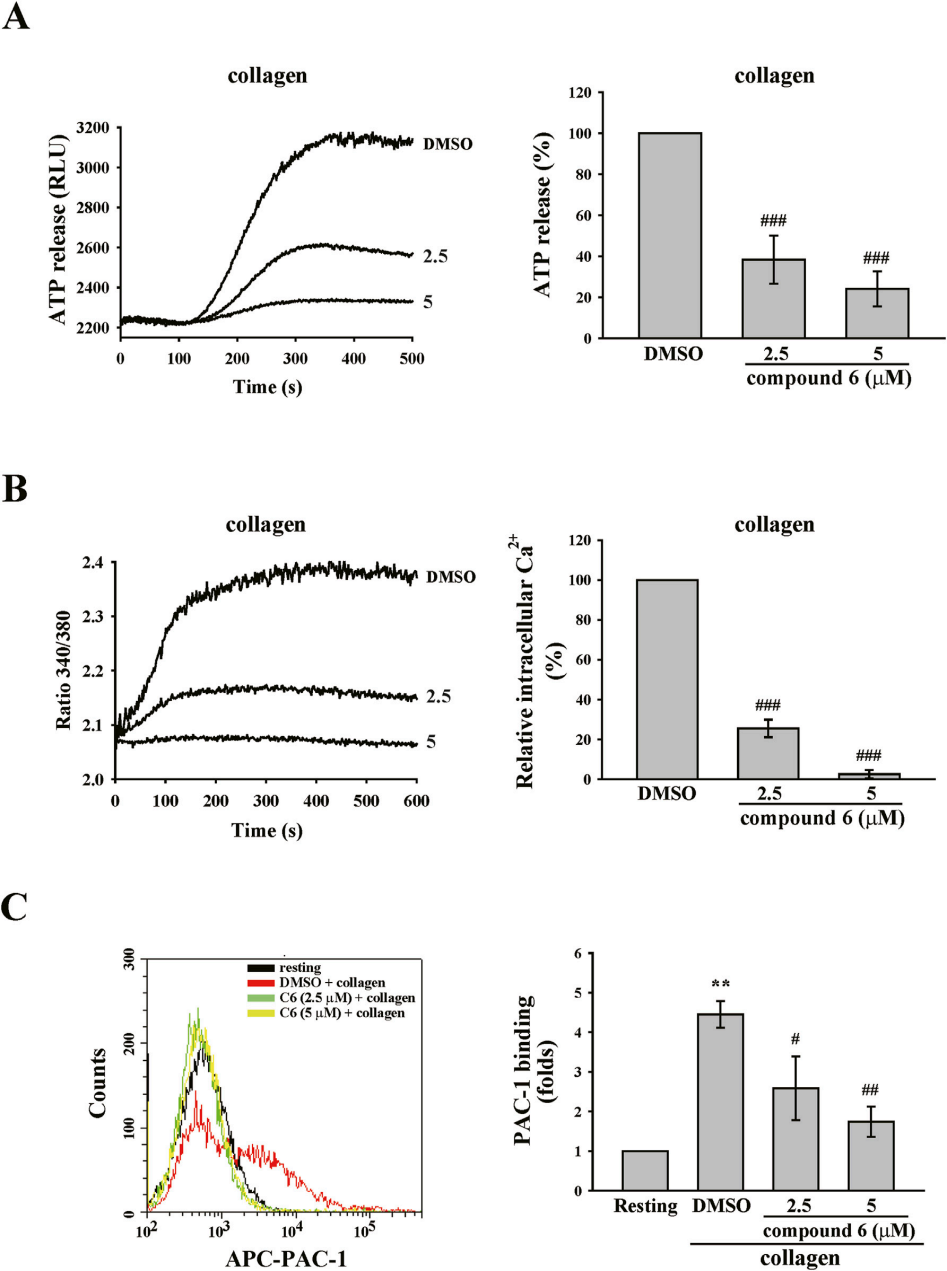
Effects of compound **6** on ATP release, calcium mobilization, and GPIIb–IIIa activation triggered by collagen in human platelets. Washed platelets (3.6 × 10^8^ cells/mL) were treated with compound **6** (**C6**, 2.5 and 5 μM) or DMSO and then stimulated with collagen (1 μg/mL) to trigger ATP release **(A)**, calcium mobilization **(B)**, and GPIIb–IIIa activation **(C)** that were detected using luciferase/luciferin, Fura-2 AM, and APC–PAC1 antibodies, respectively. Data **(A–C)** are presented as means ± SEM (*n* = 4). **p < 0.01 and ***p < 0.001 compared with the resting group. #*p* < 0.05, ##*p* < 0.01, and ###*p* < 0.001 compared with the DMSO (solvent control) group.

Platelet granule release is a crucial process for amplifying platelet activation. Calcium contributes to granule release and the inside-out activation of GPIIb–IIIa, which is responsible for the final step of platelet aggregation (Varga-Szabo et al., 2009). Thus, we observed the effect of compound **6** on such platelet activations. The data revealed that compound **6** (2.5 and 5 μM) markedly reduced collagen-triggered ATP release (Figure 8A), which indicated that compound **6** can inhibit granule release. Additionally, compound **6** (2.5 and 5 μM) could significantly reduce calcium mobilization and GPIIb–IIIa activation, as detected using Fura-2 AM and APC-PAC-1, respectively, through flow cytometry (Figures 8B,C). These results indicate that compound **6** can inhibit GPVI signaling and subsequent activation events, such as calcium mobilization, granule release, and GPIIb–IIIa activation, and eventually suppress platelet aggregation.

## 4 Discussion

In 2021, we have reported that the naphthalimide derivative compound **5** possesses antiplatelet and antithrombotic activities. However, similar to currently used clinical antiplatelet agents, compound **5** tended to affect bleeding cessation (Shih et al., 2021). In the present study, we discovered a safer and more potent naphthalimide derivative, compound **6**. We demonstrated that compound **6** is more selective in inhibiting collagen-triggered platelet activation, in part, via inhibiting GPVI. Compound **6** could reduce granule release, calcium mobilization, and GPIIb–IIIa activation, finally suppressing platelet activation and thrombus formation. Moreover, compound **6** did not affect hemostasis. These findings indicate that developing antiplatelet drugs with higher selectivity for GPVI may be safe and promising.

Naphthalimides are a class of polycyclic imides that consist of flat aromatic ring systems. Naphthalimides can intercalate with DNA and inhibit Topo II due to their planar and heteroaromatic structure. Thus, the structure of naphthalimide has been used as a core scaffold for the development of antitumor and anti-inflammatory agents (Kamal et al., 2013; Tandon et al., 2017). Moreover, the usefulness of the 1,8-naphthalimide core has been extended to more than application as an anticancer agent. For example, its derivatives possess antibacterial, antiprotozoal, antiviral, and antioxidant agents (Kamal et al., 2013). We previously reported that compound **5** also possesses antiplatelet and antithrombotic activities (Shih et al., 2021).

Although compound **6** has similar inhibitory mechanisms of platelet activation to those of compound **5**, compound **6** has better potency, selectivity, and safety than compound **5**. In comparison with compound **5**, compound **6** has an adamantane moiety. Previously, lipophilic bullet adamantane modification is used to increase the lipophilicity and stability of drugs (Wanka et al., 2013). The addition of adamantane moieties has been reported to increase the permeability of the modified compound through the blood–brain barrier (Gerzon et al., 1967; Swift et al., 1988). The add-on strategy, in which an adamantane modification is used as a functional subunit or secondary pharmacophore to enhance the activity of the primary pharmacophore, has been frequently employed (Wanka et al., 2013). For example, incorporating the adamantane moiety into neuropeptides has been reported to frequently increase the selectivity for the receptor subtype (Blommaert et al., 1993; Bellier et al., 1997). Use of the adamantane scaffold was indicated to orientate pharmacophores to positions that lead to better interactions with the target’s active site (Wanka et al., 2013). These observations revealed that adamantane modifications may provide an opportunity for original compounds to achieve more optimal selectivity and activity. In the present study, compound **6** demonstrated better selectivity and binding affinity for GPVI than compound **5** did. Unlike compound **5**, which can block thrombin- and U46619-induced platelet aggregation at high concentrations (Shih et al., 2021), compound **6** only blocked collagen-induced platelet aggregation. Moreover, compound **6** exhibited a potency that is twice as high as that of compound **5** in inhibiting collagen-induced platelet aggregation (Shih et al., 2021). These observations indicate that adamantane modification may lead naphthalimide to have increased selectivity and potency for GPVI. Additionally, the results reveal that targeting the collagen receptor GPVI can prevent thrombosis and preserve hemostasis (Mammadova-Bach et al., 2015).

GPVI plays a crucial role in maintaining vascular integrity and stabilizing arterial thrombosis and thrombus structure. Upon vascular injury, circulating platelets adhere to and become activated by collagen exposed in the extracellular matrix through GPVI. This interaction triggers the activation of Src family kinases, Fyn and Lyn, which in turn phosphorylate ITAM tyrosine residues, leading to the recruitment and activation of the tyrosine kinase Syk. This signaling cascade activates PLCγ2 and its downstream effectors, initiating platelet activation. This process is accompanied with the release of ADP and TxA_2_ that amplifies platelet activation, including integrin activation, enhancing granule content release, and promoting procoagulant platelet formation. Eventually, platelet plug forms to stop bleeding in the physiological conditions or thrombus grows in the pathological conditions (Estevez and Du, 2017; Borst and Gawaz, 2021; Mangin et al., 2023). In the present study, our data also showed that compound **6** could inhibit GPVI signaling, including the inhibition of Fyn, Lyn, and PLCγ2 phosphorylation, and subsequent granule release and GPIIbIIIa activation, finally blocking platelet activation.

However, GPVI is considered to play only a minor role in hemostasis, because previous reports have showed that patients with GPVI deficiency exhibit only mild bleeding, but their platelets fail to aggregate in response to collagen (Dutting et al., 2012). Moreover, the hemorrhagic manifestations in patients with GPVI deficiency are generally milder compared to those observed in individuals with Glanzmann’s thrombasthenia or Bernard-Soulier Syndrome, suggesting that GPVI plays a less critical role in hemostasis than GPIIbIIIa or the GPIb-IX-V complex. (Mangin et al., 2023). Moreover, an anti–GPVI-Fab fragment (glenzocimab) and a dimeric GPVI-Fc fusion protein (Revacept), which can inhibit the interaction between platelets and collagen, were reported not to interfere with normal hemostasis (Ungerer et al., 2011; Lebozec et al., 2017). Mice with genetic deletion and immunodepletion of GPVI exhibited protective effects against arterial thrombosis but moderate bleeding (Dutting et al., 2012). Thus, the hypothesis was proposed that “GPVI plays an unessential role in primary hemostasis and plug formation under physiological conditions but is important in non-hemostatic functions of the platelet (Mangin et al., 2023). Such evidence indicates that targeting GPVI may be potential therapeutic strategy for developing antiplatelet or antithrombotic drugs. Indeed, rour findings demonstrate that compound **6** selectively inhibited GPVI, effectively delaying thrombus formation in multiple murine thrombosis models. Notably, this antithrombotic effect was achieved without bleeding tendency. Although the naphthalimide derivative compound **6** did not affect hemostasis and has good safety profile, possible off-target could not be excluded. Previously, 1,8-naphthalimide pharmacophore has been reported to intercalate with DNA and inhibit topoisomerase, resulting in the inhibition of tumor cell growth and metastasis. In addition, they have also been known to exert their anticancer properties via other pathways like p53 pathway, lysosomal pathway, mitochondrial pathway (Tandon et al., 2022). Whether these pathways also affect platelet activation remains to be further defined.

In the present study, both arterial and venous thrombotic models were employed to evaluate the antithrombotic effect of compound **6**, demonstrating its potential to treat both arterial and venous thrombosis. However, compound **6** is a hydrophobic drug. Previous reports have indicated that aqueous solubility is a critical factor influencing drug absorption following oral administration, with the dissolution step often serving as the rate-limiting process in drug absorption (Krishnaiah, 2010; Bhalani et al., 2022). Therefore, if this drug is intended for clinical use in the future, its pharmacokinetics should be evaluated to confirm its bioavailability. In the case of poor bioavailability, alternative formulations—such as emulsions or nanoparticles—should be considered to enhance its absorption.

## 5 Conclusion

In conclusion, our findings indicated that the naphthalimide derivative compound **6** has antiplatelet and antithrombotic effects without affecting hemostasis because it selectively inhibits GPVI. Moreover, compound **6** may have therapeutic potential for treating patients with cardiovascular diseases.

## Data Availability

The original contributions presented in the study are included in the article/Supplementary Material, further inquiries can be directed to the corresponding author.
