# Supplementary material for: A naphthalimide derivative exerts potent antiplatelet and antithrombotic activities without a bleeding tendency
Source: Front Pharmacol. 2025 Jun 24;16:1541255. doi: 10.3389/fphar.2025.1541255 (PMC12234328; doi:10.3389/fphar.2025.1541255)
Supplement: Supplementary file 1 [file Image5.pdf]

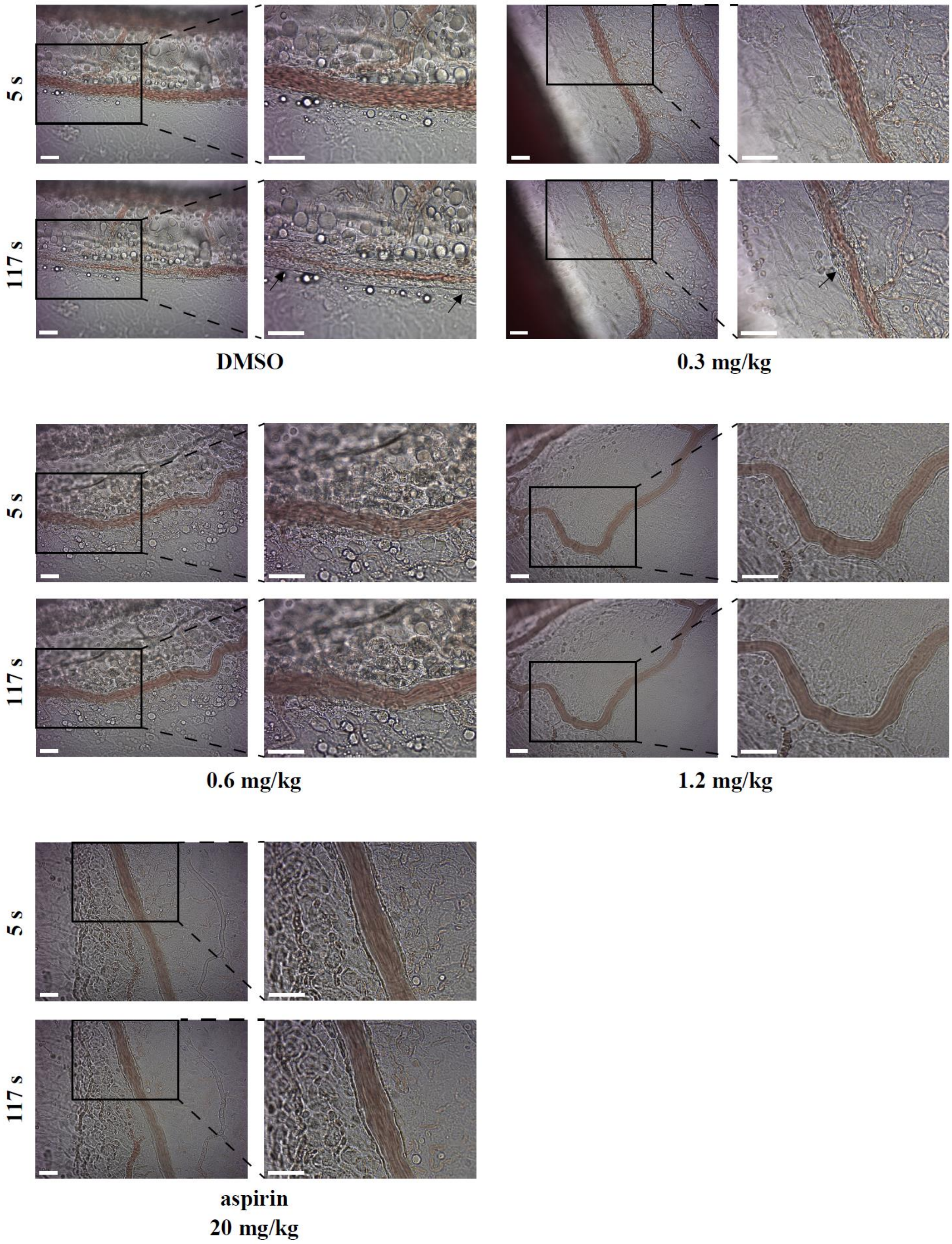

Supplementary Fig. 5. The original and enlarged images of fluorescein sodium–mediated thrombus formation in mesenteric microvessels.
