# Supplementary material for: A naphthalimide derivative exerts potent antiplatelet and antithrombotic activities without a bleeding tendency
Source: Front Pharmacol. 2025 Jun 24;16:1541255. doi: 10.3389/fphar.2025.1541255 (PMC12234328; doi:10.3389/fphar.2025.1541255)
Supplement: Supplementary file 2 [file Image6.pdf]

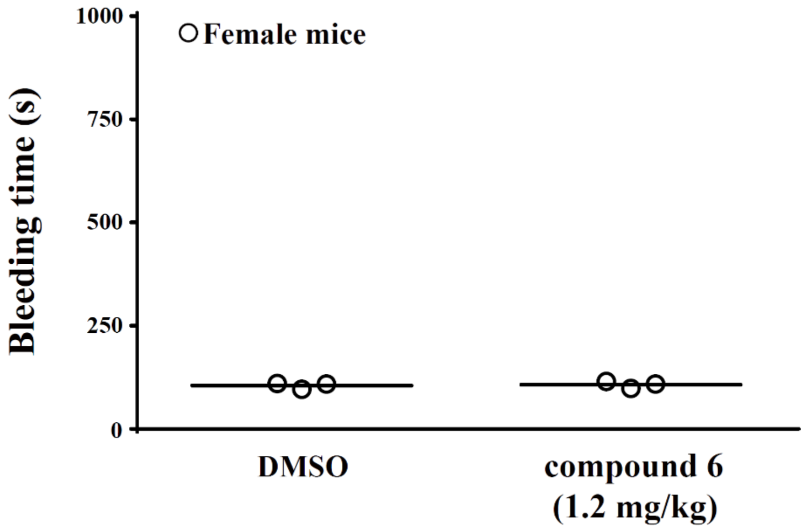

Supplementary Fig. 6. Effects of compound **6** on hemostasis in female mice. Female mice were administered with DMSO (solvent control) and compound **6** (1.2 mg/kg) (*n* = 3).
