# Supplementary material for: A naphthalimide derivative exerts potent antiplatelet and antithrombotic activities without a bleeding tendency
Source: Front Pharmacol. 2025 Jun 24;16:1541255. doi: 10.3389/fphar.2025.1541255 (PMC12234328; doi:10.3389/fphar.2025.1541255)
Supplement: Supplementary file 3 [file Image4.pdf]

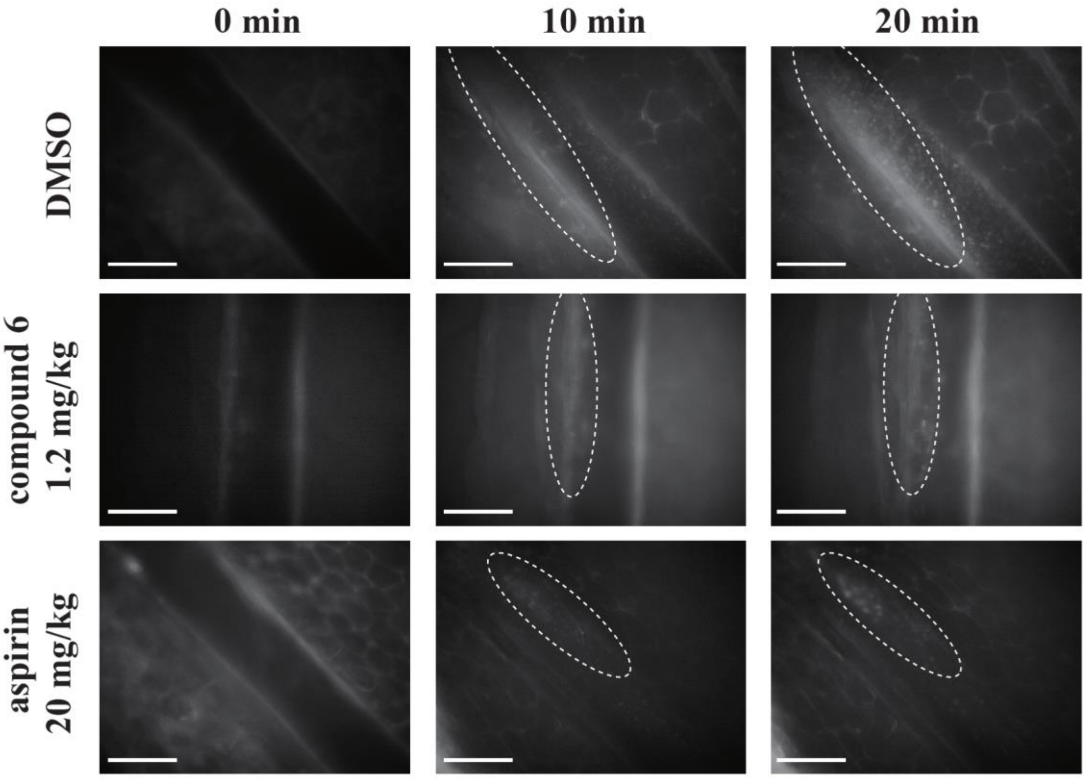

Supplementary Fig. 4. Effects of compound **6** on $FeCl_3$-induced thrombosis in mesenteric artery. Mice were intravenously administered with DMSO (solvent control), compound **6** (1.2 mg/kg), or aspirin (20 mg/kg, positive control) 10 min before $FeCl_3$ treatment. Thrombus was observed for the indicated time (0, 10, and 20 min). Scale bar = 100 μm.
