# Supplementary figures and images for: A naphthalimide derivative exerts potent antiplatelet and antithrombotic activities without a bleeding tendency
Source: Front Pharmacol. 2025 Jun 24;16:1541255. doi: 10.3389/fphar.2025.1541255 (PMC12234328; doi:10.3389/fphar.2025.1541255)

**A**

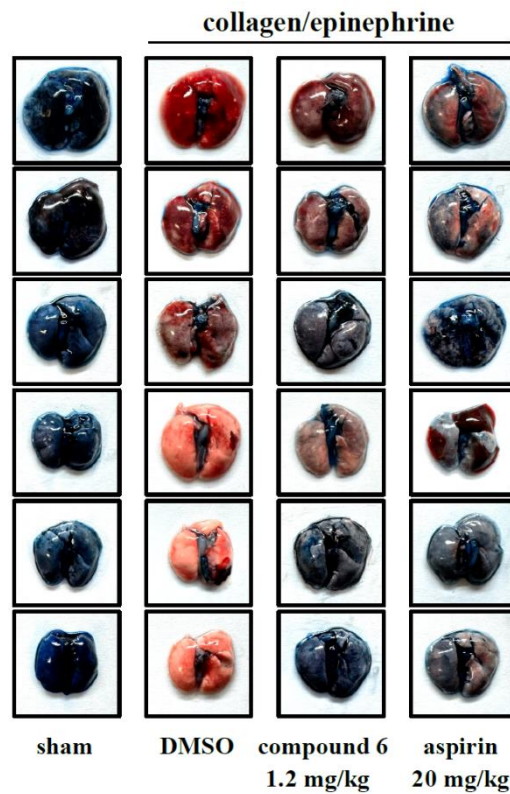

**B**

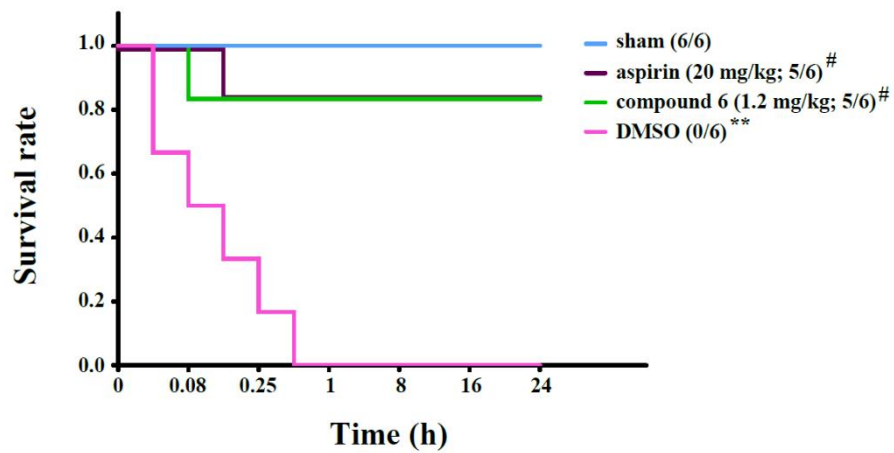

Supplement: Supplementary file 5 [file Image3.pdf]
