# Supplementary material for: A naphthalimide derivative exerts potent antiplatelet and antithrombotic activities without a bleeding tendency
Source: Front Pharmacol. 2025 Jun 24;16:1541255. doi: 10.3389/fphar.2025.1541255 (PMC12234328; doi:10.3389/fphar.2025.1541255)
Supplement: Supplementary file 6 [file Image7.pdf]

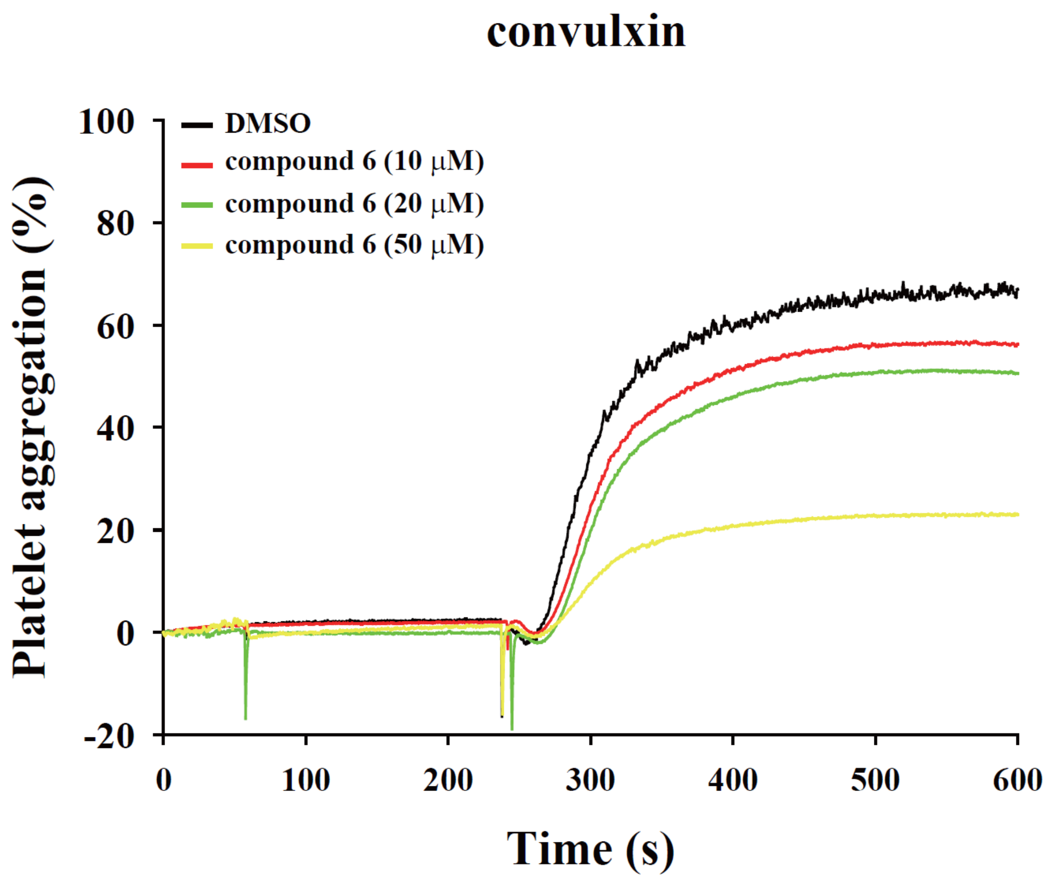

Supplementary Fig. 7. Effects of compound **6** on human platelet aggregation triggered by convulxin. Washed platelets (3.6 × 10^8 cells/mL) were treated with compound **6** (10-50 μM) or dimethyl sulfoxide (DMSO; solvent control) before adding convulxin (4 ng/mL).
