# Supplementary material for: A naphthalimide derivative exerts potent antiplatelet and antithrombotic activities without a bleeding tendency
Source: Front Pharmacol. 2025 Jun 24;16:1541255. doi: 10.3389/fphar.2025.1541255 (PMC12234328; doi:10.3389/fphar.2025.1541255)
Supplement: Supplementary file 7 [file Image1.pdf]

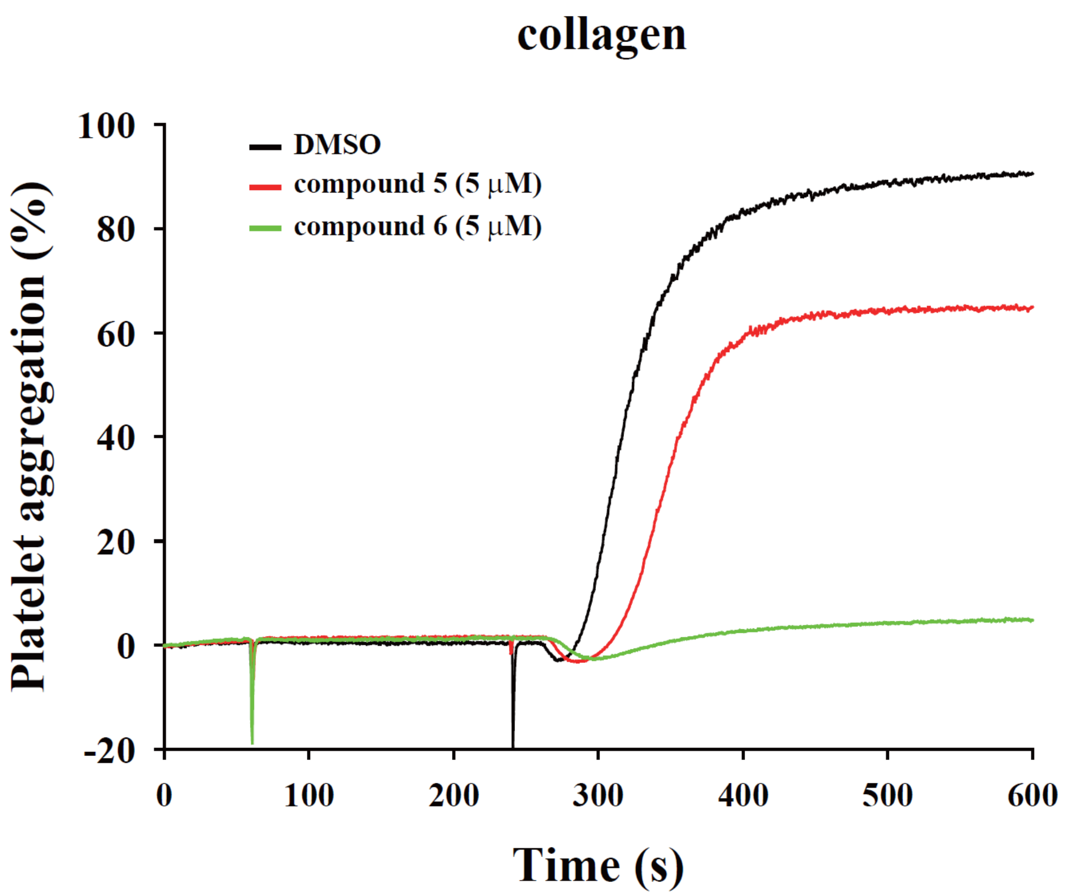

Supplementary Fig. 1. Effects of compound **5** and **6** on human platelet aggregation. Washed platelets (3.6×10^8 cells/mL) were treated with compound **5** (5 μM) and compound **6** (5 μM) or dimethyl sulfoxide (DMSO; solvent control) before adding collagen (1 μg/mL).
